# Increased unsaturated lipids underlie lipid peroxidation in synucleinopathy brain

**DOI:** 10.1186/s40478-022-01469-7

**Published:** 2022-11-14

**Authors:** YuHong Fu, Ying He, Katherine Phan, Surabhi Bhatia, Russell Pickford, Ping Wu, Nicolas Dzamko, Glenda M. Halliday, Woojin Scott Kim

**Affiliations:** 1 Brain and Mind Centre, The University of Sydney, Camperdown, NSW 2050 Australia; 2 School of Medical Sciences, The University of Sydney, Sydney, NSW Australia; 3 Bioanalytical Mass Spectrometry Facility, University of New South Wales, Sydney, NSW Australia; 4 School of Medical Sciences, University of New South Wales & Neuroscience Research Australia, Sydney, NSW Australia

**Keywords:** Lipid peroxidation, Synucleinopathies, Parkinson’s disease, Lipid aldehydes, Unsaturated lipids

## Abstract

Lipid peroxidation is a process of oxidative degradation of cellular lipids that is increasingly recognized as an important factor in the pathogenesis of neurodegenerative diseases. We were therefore interested in the manifestation of lipid peroxidation in synucleinopathies, a group of neurodegenerative diseases characterized by the central pathology of α-synuclein aggregates, including Parkinson’s disease, multiple system atrophy, dementia with Lewy bodies and Alzheimer’s disease with Lewy bodies. We assessed lipid peroxidation products, lipid aldehydes, in the amygdala, a common disease-affected region in synucleinopathies, and in the visual cortex, a disease-unaffected region. We found that the levels of lipid aldehydes were significantly increased in the amygdala, but not in the visual cortex. We hypothesized that these increases are due to increases in the abundance of unsaturated lipids, since lipid aldehydes are formed from unsaturated lipids. We undertook a comprehensive analysis of membrane lipids using liquid chromatography-mass spectrometry and found that unsaturated phosphatidylcholine, phosphatidylethanolamine, phosphatidylserine and sphingomyelin were specifically elevated in the amygdala and correlated with increases in lipid aldehydes. Furthermore, unsaturated phosphatidylethanolamine levels were associated with soluble α-synuclein. Put together, these results suggest that manifestation of lipid peroxidation is prevalent in synucleinopathies and is likely to be due to increases in unsaturated membrane lipids. Our findings underscore the importance of lipid peroxidation in α-synuclein pathology and in membrane structure maintenance.

**Supplementary Information:**

The online version contains supplementary material available at 10.1186/s40478-022-01469-7.

## Introduction

Synucleinopathies are a group of neurodegenerative diseases that have in common the central pathology of α-synuclein aggregates in the brain with evidence of α-synuclein transmission between cells [37]. Synucleinopathies include Parkinson’s disease (PD), dementia with Lewy bodies (DLB) and multiple system atrophy (MSA) with neuronal α-synuclein-positive Lewy pathologies dominating PD and DLB [45], while α-synuclein-positive glial cytoplasmic inclusions dominate MSA [21]. Alzheimer's disease with Lewy bodies (ADLB) is also considered a synucleinopathy, although it is pathologically quite distinct from other synucleinopathies in that α-synuclein aggregates present in ADLB brains are mostly restricted to the amygdala [2, 28].

An important aspect of α-synuclein biology for its oligomerization is its physiochemical interaction with lipids. α-Synuclein normally exists as a natively unfolded monomeric protein within the cytoplasm. However, when it interacts with lipids, such as phospholipid membranes, it undergoes structural changes that results in the formation of insoluble aggregates [13, 27]. It is thought that the amphipathic α-helical motif present in α-synuclein, which is similar to motifs present in lipid-binding proteins (e.g. apolipoproteins), plays a crucial role in the phospholipid binding process [41]. α-Synuclein-lipid binding, and its subsequent oligomerization, is dependent on the composition of membrane phospholipids with preferential binding to regions enriched in sphingolipids, such as sphingomyelin (SM) [5, 11, 20, 27, 32]. It is also dependent on asymmetry of the phospholipid bilayer containing the phospholipids phosphatidylcholine (PC), phosphatidylethanolamine (PE) and phosphatidylserine (PS) [12, 18, 29].

The integrity and function of membrane phospholipids are detrimentally affected by lipid peroxidation [34], which is a process of oxidative degradation of lipids caused by reactive oxygen species (ROS) or by via enzymatic routes involving enzymes, such as 15-lipoxygenases and aldehyde dehydrogenases [10, 17]. ROS specifically targets carbon–carbon double bonds in unsaturated fatty acids. The brain is particularly susceptible to ROS due to its high content of unsaturated fatty acids in membranes and high oxygen consumption [44]. Lipid peroxidation causes a direct degradation of membrane phospholipids resulting in membrane dysfunction. It also results in the formation of lipid aldehydes, such as acrolein and malondialdehyde, which are highly reactive and toxic as they bind to cellular proteins and annul their function. Growing evidence indicates that toxic products formed from lipid peroxidation contribute to the pathogenesis of most, if not all, neurodegenerative diseases, as well as occurring in normal ageing [36, 39].

Here, we investigated manifestation of lipid peroxidation across multiple synucleinopathies, namely PD, DLB, MSA and ADLB. Specifically, we measured lipid aldehydes, acrolein and malondialdehyde in the common disease-affected region amygdala. We also investigated the possible cause of the elevation of lipid aldehydes in synucleinopathies by analyzing the abundance of unsaturated PC, PE, PS and SM in the amygdala.

## Materials and methods

### Human brain tissues

Frozen post-mortem brain tissue samples were obtained from Sydney Brain Bank and NSW Brain Tissue Resource Centre. Ethical approvals were acquired from the human research ethics committees of University of New South Wales (approval number: HC16568) and the University of Sydney (approval number: 2020/707). Frozen samples from the amygdala and the visual cortex from 8 PD, 5 DLB, 8 MSA and 7 ADLB cases and 10 controls without neurological, psychiatric or neuropathological diagnoses were used in this study (Additional file 1: Table S1).

### Chemicals and materials

Lipids were extracted using chloroform or methyl-t-butyl ether, methanol and isopropanol (Sigma Aldrich, St. Louis, MO, USA) and ultrapure water (Millipore). All solvents used were HPLC grade or higher. Glass pipettes and tubes were used wherever possible and the use of plasticware was minimized during lipid extraction to avoid contamination of samples. Glass tubes and glass transfer pipettes were purchased from Sigma and VWR. Lipid internal standards (ISTDs) were purchased from Avanti Polar Lipids Inc. (Alabaster, AL, USA). These include phosphatidylcholine (19:0), sphingomyelin (12:0), phosphatidylethanolamine (17:0), phosphatidylglycerol (17:0), phosphatidylserine (17:0), phosphatidic acid (17:0), ceramide (d18:1, 12:0), diglyceride (1,3 18:0 d5), cholesteryl ester (19:0), monoglyceride (17:0), triglyceride mix d5 (Avanti Code LM-6000), diglyceride mix d5 (Avanti Code LM-6001), phosphatidylinositol (17:0 14:1), C12 GluCer, C12 sulfatide, C17 ceramide, C17 sphingosine, C17 S1P, C12 C1P, D3 C20 fatty acid, and C12 LacCer. Lipid internal standards were prepared as a mixture at 10 pmol/µl in methyl-tert butyl ether and methanol (MTBE:methanol, 1:1 v/v).

### Immunohistochemistry

Formalin-fixed paraffin-embedded sections were deparaffinized with xylene and then rehydrated with gradient ethanol and water. Antigen retrieval was conducted with 70% formic acid followed by heating in a pressure cooker (Aptum Bio Retriever 2100, Aptum Biologics Ltd, UK). Endogenous peroxidase activity was quenched with 1% H_2_O_2_ in 50% ethanol for 30 min and then washed in distilled water. Sections were further blocked with 5% normal horse serum prior to incubation in medium containing primary antibody phospho-S129 (Abcam Ab51253, 1:500) or O2 (Creativebiolab TAB-0748CLV, 1:200) at 4 °C for 2 days. Sections were washed and incubated with either alkaline phosphatase (AP)-anti-mouse or horseradish peroxidase (HRP)-anti-rabbit secondary polymer antibodies (VECTOR, ImmPRESS, cat.# MP-5402 & MP-7401). The immunoreactive color was developed with ImmPACT Vector Red AP Substrate (VECTOR, SK-5105) or ImmPACT DAB EqV Peroxidase (HRP) Substrate (VECTOR, SK-4103) respectively, prior to counterstaining with hematoxylin. Sections were then dehydrated with gradient ethanol and cleared with xylene prior to coverslipping with D.P.X. Images were obtained at × 20 magnification using an Olympus slide scanner (VS120).

### Protein extraction

Tris-buffered saline (TBS) and SDS-soluble proteins were serially extracted from 100 mg of fresh-frozen brain tissues, as previously described [31]. Briefly, tissues were homogenized in ten volumes of TBS homogenization buffer (20 mM Tris, 150 mM NaCl, pH 7.4, 5 mM EDTA, 0.02% sodium azide) containing protease inhibitor cocktail (Roche) using Qiagen TissueLyser (3 × 30 s, 30 Hz cycles), followed by centrifugation at 100,000 g for 1 h at 4 °C, with supernatant collected as TBS-soluble fraction. The pellet was resuspended in SDS solubilization buffer (TBS homogenization buffer containing 5% SDS) using 3 × 30 s, 30 Hz cycles with TissueLyser, and centrifuged at 100,000 g for 30 min at 25 °C, with supernatant collected as SDS-soluble fraction. Protein concentration was measured using a bicinchoninic acid assay (Pierce BCA Protein Assay Kit) following the manufacturer’s instructions.

### Western blotting and ELISA

Protein lysates (10 µg) were heated with sample buffer (3.2% SDS, 32% glycerol, 0.16% bromophenol blue, 100 mM Tris–HCl, pH 6.8, 8% 2-mercaptoethanol). They were then electrophoresed on Criterion Stain-free 4–20% SDS-PAGE gels (Bio-Rad) and transferred onto nitrocellulose membranes at 100 V for 30 min. The membranes were blocked with TBS containing 5% nonfat dry milk and probed with α-synuclein antibody (1:1000, BD Biosciences, 610,787), NFL antibody (1:2000, Cell Signaling, 2835S) or acrolein antibody (1:1000, NOVUS, NB200-556) overnight at 4 °C. They were then washed three times in TBS containing 0.1% Tween 20 and incubated with horseradish peroxidase-conjugated secondary antibody for 2 h at room temperature. Signals were detected using enhanced chemiluminescence and Gel Doc System (Bio-Rad). The blots were stripped and probed for housekeeper proteins tubulin or GAPDH. The signal intensity was quantified using Image Lab (Bio-Rad) and NIH ImageJ software (v1.45 s). ELISA of α-synuclein (Legend Max Cat no: 844101) was carried out following the manufacturer’s protocol. ELISA of neurofilament light (Novus Cat no: NBP2-81,184) was carried out following the manufacturer’s protocol.

### RNA extraction and quantitative PCR

RNA was isolated using TRIzol reagent (Invitrogen) following the manufacturer’s protocol as previously described [36]. All procedures were carried out using RNase-free reagents and consumables. One microgram of RNA was reverse transcribed into cDNA using Moloney-murine leukemia virus (M-MLV) reverse transcriptase and random primers (Promega, Madison, Wisconsin, USA) in 20 μl reaction volume. Quantitative PCR (qPCR) assays were carried out using a Mastercycler ep realplex S (Eppendorf, Sydney, Australia) and the fluorescent dye SYBR Green (Bio-Rad), following the manufacturer’s protocol. Briefly, each reaction (20 μl) contained 1 × mastermix, 5 pmol of primers and 1 μl of cDNA template. Amplification was carried out with 40 cycles of 94 °C for 15 s and 60 °C for 1 min. Gene expression was normalized to the geometric mean of three housekeeper genes, GAPDH, β-actin and PPIA. A no-template control was included for each PCR amplification assay. The level of expression for each gene was calculated using the comparative threshold cycle (Ct) value method using the formula 2^−ΔΔCt^ (where ΔΔCt = ΔCt sample – ΔCt reference).

### Malondialdehyde assay

Malondialdehyde was measured using Lipid Peroxidation MDA Assay Kit (Abcam, cat. # ab118970) following the manufacturer’s protocol. Briefly, TBA solution was added into each vial containing samples or standards and incubated at 95 °C for 1 h. Signal at 532 nm was read using CLARIOstar plate reader (BMG Labtech).

### Lipid extraction

Brain tissue lipid extraction was based on the Matyash method [30]. Briefly, 10 mg of fresh-frozen brain tissues were homogenized in methanol containing 0.01% BHT (300 µl) using a Qiagen TissueLyser (3 × 30 s, 30 Hz cycles). The homogenates were transferred to glass tubes, as well as the methanol (430 µl) wash of the beads. MTBE (2.42 ml) was added and the mixture vortexed and incubated for 30 min at room temperature. Water (600 µl) was added and the mixture vortexed and centrifuged at 1000 g for 10 min. The upper phase was transferred to a new glass tube using a glass Pasteur pipette. The lower phase was re-extracted using MTBE/MeOH/water (10:3:2.5). The preparation was dried under nitrogen gas. Dried lipid samples were reconstituted in 100 µl of chloroform/methanol (1:1) and stored at − 80℃.

### Liquid chromatography—mass spectrometry

Lipid extracts (10 μl) were analyzed using a Q-Exactive Plus Mass Spectrometer coupled to a U3000 UPLC system (ThermoFisher Scientific). Chromatography was performed at 60℃ on a Waters CSH C18 UHPLC column 2.1 × 100 mm, 1.8 μM with VanGuard guard column. Solvent A was 6:4 acetonitrile:water and Solvent B was 1:9 acetonitrile:isopropanol, both with 10 mM ammonium formate and 0.1% formic acid. Lipids were chromatographed according to the method of Castro-Perez et al. [8]. Briefly, a 30 min gradient running from 30 to 100% of solvent B was performed, eluting lipids in order of hydrophobicity. Column eluate was directed into the electrospray ionization source of the mass spectrometer where a HESI probe was employed. Source parameters were broadly optimized on a range of lipid standards prior to the analysis. The mass spectrometer was run in data dependent acquisition mode. A survey scan over the mass range 200–1200 at resolution 70 K was followed by 10 data dependent MS/MS scans on the most intense ions in the survey at 15 K resolution. Dynamic exclusion was used to improve the number of ions targeted. Cycle time was approximately 1 s. Samples were run in both positive and negative polarities. The samples were run in a random order (generated using Microsoft Excel). This is important to avoid batch effects/changing instrument performance effects. Data were analysed in LipidSearch software 4.1.16. Data were searched against the standard Lipidsearch database with all common mammalian lipid classes included. The search results were then grouped according to sample type and aligned for differential analysis. Aligned data (containing lipid identity, retention time, peak area etc.) were exported to Excel software. Relative abundance of lipids was obtained from peak areas normalized to internal standards.

### Statistical analysis

Statistical analyses were performed using SPSS Statistics software (IBM, Chicago, Illinois) as previously described [36]. Multivariate analyses (general linear model) covarying for age and sex were used to determine differences in lipid levels in the synucleinopathies and control groups with posthoc statistical significance set at *p* < 0.05. Pearson’s correlations were used to determine if changes in measurements were associated with each other with statistical significance set at *p* < 0.05. Graphs were generated using GraphPad Prism 9.

## Results

### Verification of synucleinopathies

**Fig. 1:**
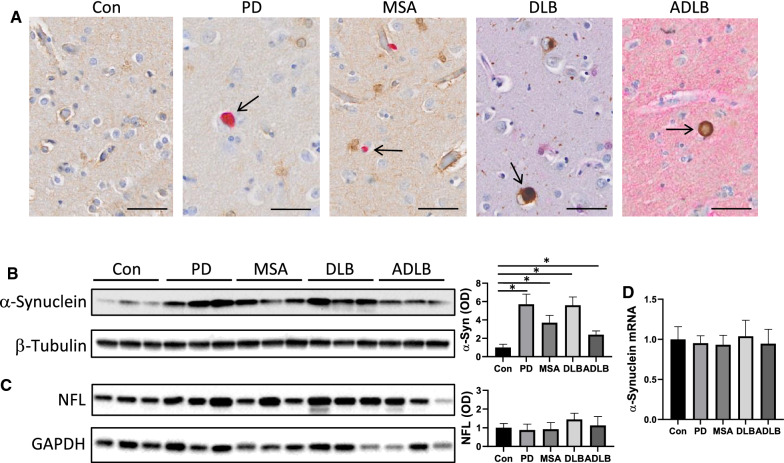
Presence of α-synuclein pathology in synucleinopathies. **a** Representative images of immunohistochemistry of α-synuclein aggregates (arrowed) in the amygdala of Parkinson’s disease (PD), multiple system atrophy (MSA), dementia with Lewy bodies (DLB) and Alzheimer’s disease with Lewy bodies (ADLB) and absent in controls (Con); scale bars = 50 µm. **b** Western blotting of insoluble fraction of α-synuclein in the amygdala and optical density (OD) measurement of the protein bands normalized to β-tubulin. **c** Western blotting of neurofilament light (NFL) in the amygdala and OD measurement of the protein bands normalized to GAPDH. **d** mRNA expression of α-synuclein in the amygdala. Data represent mean and SE as error bars, *P < 0.05

Lipid peroxidation is increasingly recognized as a significant contributing factor in the pathogenesis of neurodegenerative diseases. We were therefore interested in the manifestation of lipid peroxidation across synucleinopathies (PD, DLB, MSA and ADLB). Firstly, we confirmed the presence of α-synuclein pathology in these synucleinopathies by immunostaining (Fig. 1a) and by western blotting of the amygdala (Fig. 1b). As a gauge of neurodegeneration, we measured neurofilament light (NFL) in the same tissues by western blotting (Fig. 1c) and ELISA (Additional file 1: Fig. S1), and found that it was unaltered in the synucleinopathies compared controls, suggesting that the neuronal integrity of these tissues was still intact. We also showed that α-synuclein mRNA expression in the synucleinopathies was unaltered in the amygdala (Fig. 1d), indicating that α-synuclein aggregates present in the synucleinopathies was due to increased α-synuclein accumulation rather than increased α-synuclein production.

### Increases in lipid aldehydes in synucleinopathies

**Fig. 2:**
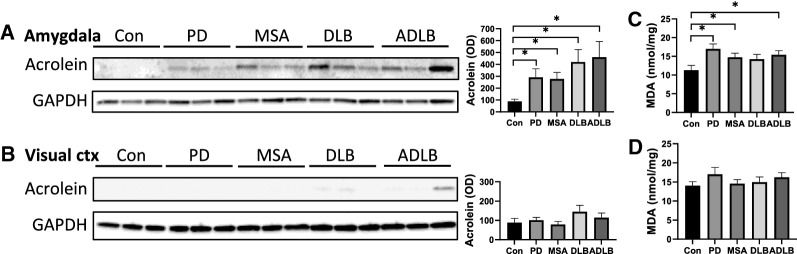
Analysis of lipid peroxidation in synucleinopathies. Western blotting of acrolein-conjugated proteins in the amygdala (**a**) and the visual cortex (**b**), and optical density (OD) measurement of the protein bands normalized to GAPDH. A colorimetric measurement of malondialdehyde (MDA) in the amygdala (**c**) and the visual cortex (**d**). Data represent mean and SE as error bars, *P < 0.05

Lipid peroxidation results in the formation of lipid aldehydes, and the major lipid aldehydes in the brain are acrolein and malondialdehyde (MDA). Acrolein and MDA are highly reactive and readily form conjugates with cellular proteins, annulling their function, and therefore are indirect indicators of lipid peroxidation. To assess the degree of lipid peroxidation, we measured acrolein-conjugated proteins by western blotting, and MDA levels, by colorimetric assay, in the amygdala. We also measured the two aldehydes in the visual cortex, which is largely unaffected in synucleinopathies and can be considered as an unaffected control region. We found significant increases in acrolein-conjugated proteins in the amygdala in all four synucleinopathies (Fig. 2a), but no significant changes were observed in the visual cortex (Fig. 2b). MDA levels in the amygdala were also significantly increased in PD, MSA and ADLB with non-significant increases in DLB (Fig. 2c). Again there were no significant changes in the visual cortex (Fig. 2d). These results suggest that lipid peroxidation occurs in the brain of synucleinopathies in a region-specific manner.

### Increases in unsaturated lipids in synucleinopathies

**Fig. 3:**
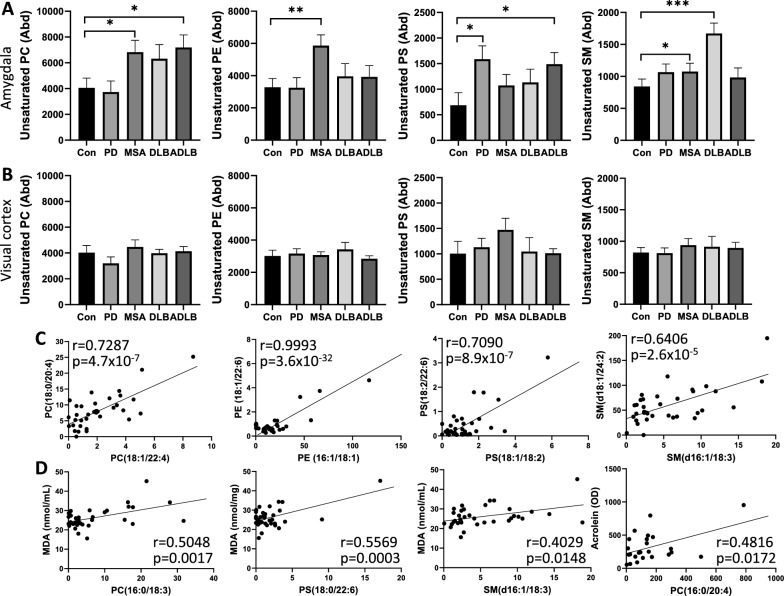
Measurement of unsaturated lipids in synucleinopathies using liquid chromatography-mass spectrometry. **a** Abundance (Abd) of unsaturated phosphatidylcholine (PC), unsaturated phosphatidylethanolamine (PE), unsaturated phosphatidylserine (PS) and unsaturated sphingomyelin (SM) in the amygdala; data represent mean and SE as error bars, *P < 0.05, **P < 0.01, ***P < 0.005. **b** Abundance of unsaturated lipids in the visual cortex. **c** Pearson’s correlation of unsaturated lipid species within each lipid class. **d** Pearson’s correlation of unsaturated lipid species with malondialdehyde (MDA) and acrolein levels in the amygdala

Since lipid aldehydes are formed from unsaturated lipids, we hypothesized that increases in lipid aldehydes in synucleinopathies are due to increases in the abundance of unsaturated lipids. Unsaturated lipids contain fatty acids with one or more carbon–carbon double bonds and these bonds are the site for the peroxidation reaction. Therefore, the greater the abundance of unsaturated lipids the greater the susceptibility to lipid peroxidation. We undertook a comprehensive analysis of lipids in the amygdala and visual cortex using liquid chromatography-mass spectrometry and the LipidSearch software. The statistics were performed using SPSS Statistics software in the multivariate analysis (general linear model) mode covarying for age and sex. We assessed four major membrane lipid classes, phosphatidylcholine (PC), phosphatidylethanolamine (PE), phosphatidylserine (PS) and sphingomyelin (SM) in each of the synucleinopathies and found elevated levels of unsaturated PC in MSA and ADLB, unsaturated PE in MSA, unsaturated PS in PD and ADLB, and unsaturated SM in MSA and DLB in the amygdala (Fig. 3a). Notably, three of the four unsaturated lipids were elevated in MSA amygdala. In contrast, none of the unsaturated lipids were elevated in the visual cortex in any of the synucleinopathies (Fig. 3b). To further understand the changes in unsaturated lipids in the amygdala, we examined lipid species and found that they were strongly correlated with one another in each of the classes (Fig. 3c), suggesting that the elevation of unsaturated lipids was universal. Importantly, specific unsaturated lipid species were correlated to MDA and acrolein levels in the amygdala (Fig. 3d).

### Correlation between unsaturated PE and soluble α-synuclein

**Fig. 4:**
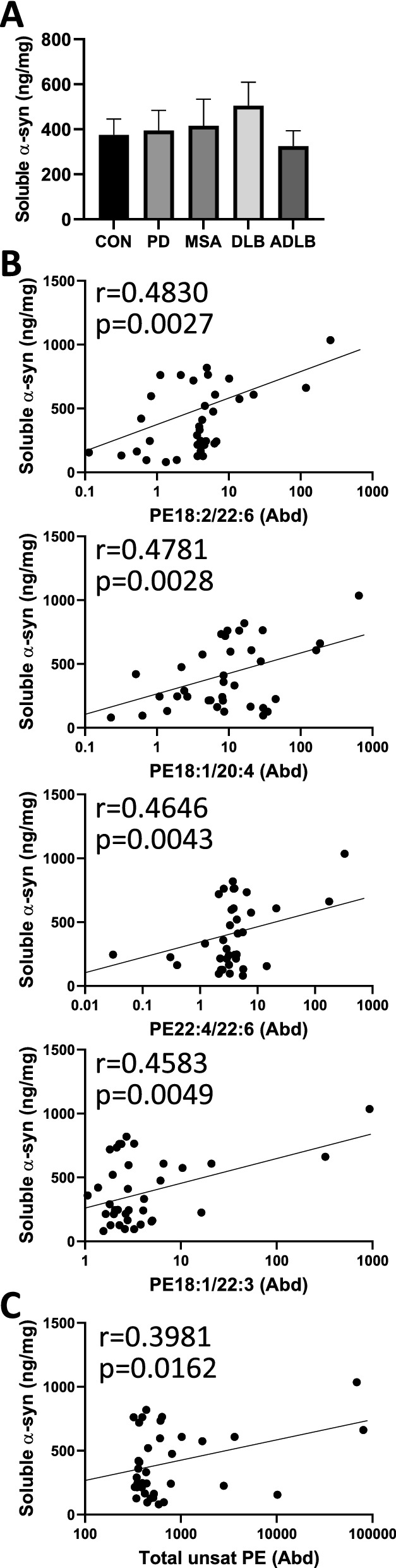
Soluble α-synuclein is associated with unsaturated phosphatidylethanolamine (PE). **a** ELISA measurement of soluble α-synuclein in the amygdala; data represent mean and SE as error bars. Pearson’s correlation of soluble α-synuclein with **b** unsaturated PE species and **c** the total unsaturated PE class in the amygdala

To further understand the effect of unsaturated lipids in synucleinopathies, we assessed the relationship between unsaturated lipids and α-synuclein, the major pathogenic protein in synucleinopathies. We measured soluble α-synuclein in the amygdala by ELISA and found no significant changes in any of the synucleinopathies compared to controls (Fig. 4a), which is consistent with the α-synuclein mRNA expression results (Fig. 1d). α-Synuclein levels, however, were significantly correlated to unsaturated PE species (Fig. 4b) and to the total unsaturated PE class (Fig. 4c). No significant correlation was observed between α-synuclein levels and the other lipid classes (Additional file 1: Fig. S2).

## Discussion

In the current study we investigated manifestation of lipid peroxidation in the brain of synucleinopathies that include PD, DLB, MSA and ADLB. We demonstrated that lipid aldehydes, acrolein and MDA, were elevated across synucleinopathies, specifically in the common disease-affected region, amygdala (Fig. 2), and that these increases were associated with increases in specific unsaturated lipids (Fig. 3). Unsaturated lipids of at least one lipid class (PC, PE, PS or SM) were elevated in the amygdala of each of the synucleinopathies, with three lipid classes elevated in MSA.

Consistent with our findings, other lipid aldehydes have been shown to be elevated in the brain of neurodegenerative diseases in a region-specific manner. For example, lipid aldehydes 4-hydroxy-2-nonenal (HNE) and 4-oxo-2-nonenal (ONE) are elevated in the substantia nigra of PD brain [16], particularly in the nigral neurons [51]. Again, HNE levels are elevated in the frontal cortex, but not in the cerebellum, of Alzheimer’s disease (AD) [40]. Interestingly, MDA, the lipid aldehyde that was elevated in PD, MSA and ADLB (Fig. 2c), has been shown to form deposits associated with lipofuscin in AD hippocampal neurons, particularly in the CA4 region [15]. MDA also colocalizes with senile plaques and neurofibrillary tangles in AD [15].

We observed increases in the abundance of unsaturated lipids with various fatty acid chain length and number of carbon–carbon double bonds, including fatty acids with 22 carbon atoms and 6 double bonds (22:6). Typically, numerous numbers of different lipid species are present in brain tissues, however the function of most of these brain lipid species is unknown. One unsaturated fatty acid that is of interest in the context of neurodegeneration is docosahexaenoic acid (DHA; 22:6) [4]. The brain is particularly enriched in DHA [9], and increases in DHA levels in PD and DLB brain are thought to contribute to the oligomerization of α-synuclein and subsequent neuronal damage [43]. The detrimental impact of increases in DHA levels is supported by the fact that DHA can form covalent bonds with α-synuclein [14]. The interaction of HNE, one of main products of DHA peroxidation, with α-synuclein has been shown to promote the formation of toxic α-synuclein oligomers [38, 42]. ONE has also been shown to promote the oligomerization of monomeric α-synuclein [6, 33], and enhance the stability and yield of α-synuclein oligomers [1]. Consistent with these findings, increases in unsaturated lipids are associated with increases in α-synuclein toxicity in neurons [19]. Furthermore, when unsaturated lipids are reduced by an inhibition of stearoyl-CoA desaturase (enzyme that converts saturated fatty acids into unsaturated fatty acids), α-synuclein toxicity in human-derived neurons is reduced [47].

Under pathological conditions, such as in synucleinopathies, α-synuclein is known to undergo conformational changes that enhances the formation of oligomers. This process is thought to be initiated when α-synuclein binds and interacts with lipid membranes. In vitro studies showed that α-synuclein monomers, isolated from primary neurons, readily bound to lipid membranes and formed oligomers [13, 27]. An amphipathic α-helical motif present in α-synuclein, similar to those present in lipid-binding proteins, is thought to facilitate the lipid membrane binding process [41]. The binding/interaction of α-synuclein to membranes and subsequent oligomerization is also dependent on the lipid composition of membranes [11, 22, 27] with preferential binding to regions of membranes that are enriched in sphingolipids [20].

We found that unsaturated PE levels were strongly correlated with soluble α-synuclein in the amygdala. This is an interesting result in light of the observation that α-synuclein accumulation is modulated by cellular PE levels [48]. In yeast and worm models of PD, decreases in the level of total PE (not unsaturated PE) resulted in increased accumulation of α-synuclein foci resembling α-synuclein deposits [48]. The role of PE is particularly important in the brain as it makes up ~ 45% of brain phospholipids [46]. A study using magnetic resonance spectroscopic imaging showed that the level of total PE (not unsaturated PE) in the putamen was decreased in brains of early (Hoehn and Yahr stages I/II) PD patients, but not in advanced (Hoehn and Yahr stages III/IV) PD patients [24]. In another study, the presence of PE in phospholipid vesicles significantly augmented the binding of soluble α-synuclein to the membrane and resulted in extensive bilayer disruption and the formation of fibrils [25]. These and other studies that implicate PE in α-synuclein pathology, however, do not stipulate whether the lipids are saturated or unsaturated, nor the identity of the lipid species involved, and therefore comparative analyses of results from different studies are difficult.

MSA had the greatest and most significant increase in unsaturated lipids out of the four synucleinopathies. MSA is the most rapidly developing and devastating disease with a broader distribution of α-synuclein pathology compared to the other three synucleinopathies [35, 49]. Another key difference between MSA and the other three synucleinopathies is that the principal cell type involved is oligodendrocytes rather than neurons. Oligodendrocytes contain the specialized membranous myelin, which is highly enriched in unsaturated lipids, and therefore susceptible to greater lipid peroxidation. Future studies could investigate the degree of lipid peroxidation in oligodendrocytes compared to neurons and their toxic effect in these two cell types in MSA. Interestingly, in multiple sclerosis, which is a chronic inflammatory disease affecting oligodendrocytes, the levels of MDA and other lipid aldehydes are increased in oligodendrocytes [23].

We observed that unsaturated PS levels were elevated in the amygdala of PD and ADLB. PS is an integral membrane phospholipid that allows asymmetrical membrane curvature. Normally, PS is sequestered to the inner leaflet of the lipid bilayer (cytosolic) in an asymmetric manner. However, lipid peroxidation of PS causes changes to the membrane structure by a redistribution of PS in the membrane. Collapse of lipid asymmetry and exposure of PS on the cell surface initiates a number of pathological changes. One such change is the initiation of early apoptotic events and consequent phagocytosis of targeted cells [50]. Lipid peroxidation of PS has been shown to be important for PS egression to the cell surface in apoptotic cells [26]. Furthermore, exposure of PS to the cell surface altered the activity of proteins embedded in membranes, such as receptors and transporters [3]. In AD brain, the lipid aldehyde acrolein altered the asymmetric distribution of PS in the membrane [7].

In summary, we have revealed that lipid peroxidation is prevalent in synucleinopathies and that increased levels of lipid aldehydes are likely to be due to increased levels of unsaturated lipids in membranes. These changes were however observed in postmortem tissues, i.e. late stages of disease, and therefore could be possibly compensatory effects, rather than pathogenic impact, although the NFL levels were not altered in the synucleinopathies compared controls, suggesting that the neuronal integrity of these tissues was still intact. Nevertheless, our findings underscore the importance of lipid peroxidation in α-synuclein pathology and in the maintenance of membrane structure and cellular homeostasis.

## Supplementary Information

**Additional file 1:** Synucleinopathy brain data.

## Data Availability

Lipidomics raw data were generated at Bioanalytical Mass Spectrometry Facility, University of New South Wales. Derived data supporting the findings of this study are available from the corresponding author, upon reasonable request. Other patient data cannot be made publicly available because the ethical approval and the informed consent from the patients included in this study did not cover placing the data into publicly open repositories.
