# Supplementary material for: Increased unsaturated lipids underlie lipid peroxidation in synucleinopathy brain
Source: Acta Neuropathol Commun. 2022 Nov 14;10:165. doi: 10.1186/s40478-022-01469-7 (PMC9664712; doi:10.1186/s40478-022-01469-7)
Supplement: Supplementary file 1 — Additional file 1: Synucleinopathy brain data. [file 40478_2022_1469_MOESM1_ESM.docx]

**Supplementary Table 1** Demographic information of cases used in this study

| **ID** | **Case** | **Age (y)** | **Sex** | **PMI (h)** | **Dis dur (y)** |
| --- | --- | --- | --- | --- | --- |
| 1 | PD | 79 | Male | 42 | 17 |
| 2 | PD | 82 | Male | 19 | 22 |
| 3 | PD | 84 | Male | 5 | 12 |
| 4 | PD | 79 | Male | 17 | 7 |
| 5 | PD | 81 | Female | 29 | 22 |
| 6 | PD | 82 | Female | 9 | 8 |
| 7 | PD | 73 | Male | 20 | 13 |
| 8 | PD | 82 | Male | 22 | 7 |
| 9 | MSA | 82 | Male | 8 | 7 |
| 10 | MSA | 61 | Male | 7 | 2 |
| 11 | MSA | 62 | Male | 31 | 10 |
| 12 | MSA | 71 | Female | 19 | 6 |
| 13 | MSA | 72 | Female | 10 | 5 |
| 14 | MSA | 74 | Female | 18 | 7 |
| 15 | MSA | 69 | Male | 41 | 8 |
| 16 | MSA | 74 | Male | 16 | 13 |
| 17 | DLB | 69 | Male | 28 | 3 |
| 18 | DLB | 78 | Male | 3 | 7 |
| 19 | DLB | 74 | Male | 16 | 8 |
| 20 | DLB | 83 | Male | 7 | 11 |
| 21 | DLB | 92 | Male | 46 | 8 |
| 22 | ADLB | 78 | Male | 18 | 4 |
| 23 | ADLB | 90 | Male | 5 | 7 |
| 24 | ADLB | 70 | Male | 8 | 1 |
| 25 | ADLB | 73 | Female | 7 | 5 |
| 26 | ADLB | 73 | Male | 37 | 8 |
| 27 | ADLB | 82 | Female | 3 | 15 |
| 28 | ADLB | 82 | Male | 7 | 7 |
| 29 | Control | 94 | Male | 24 | N/A |
| 30 | Control | 87 | Female | 24 | N/A |
| 31 | Control | 89 | Male | 22 | N/A |
| 32 | Control | 84 | Male | 9 | N/A |
| 33 | Control | 60 | Male | 25 | N/A |
| 34 | Control | 72 | Female | 25 | N/A |
| 35 | Control | 80 | Male | 12 | N/A |
| 36 | Control | 67 | Male | 29 | N/A |
| 37 | Control | 81 | Female | 35 | N/A |
| 38 | Control | 75 | Male | 34 | N/A |

**Supplementary Figure 1**

Measurement of neurofilament light (NFL) in the amygdala using ELISA.

**Supplementary Figure 2**

Pearson’s correlation of soluble α-synuclein with unsaturated phosphatidylcholine (PC), phosphatidylserine (PS) and sphingomyelin (SM) in the amygdala.
